# The Impact of Clinical Trial Quality Assurance on Outcome in Head and Neck Radiotherapy Treatment

**DOI:** 10.3389/fonc.2019.00792

**Published:** 2019-08-21

**Authors:** Haoyu Zhong, Kuo Men, Jiazhou Wang, Johan van Soest, David Rosenthal, Andre Dekker, Zhen Zhang, Ying Xiao

**Affiliations:** ^1^Department of Radiation Oncology, University of Pennsylvania, Philadelphia, PA, United States; ^2^National Cancer Center/National Clinical Research Center for Cancer/Cancer Hospital, Chinese Academy of Medical Sciences and Peking Union Medical College, Beijing, China; ^3^Fudan University Shanghai Cancer Center, Shanghai, China; ^4^Maastro Clinic, Maastricht, Netherlands; ^5^MD Anderson Cancer Center, Houston, TX, United States

**Keywords:** radiotherapy, quality assurance, treatment outcomes, clinical trial, contour, dose

## Abstract

**Purpose:** To investigate the impact of radiation treatment quality assurance (RTQA) on treatment outcomes in a phase III trial for advanced head and neck cancer.

**Materials and Methods:** A total of 767 patients from NRG/RTOG 0522 were included in this study. The contours of target volume (TV) and organ at risk (OAR), and dose-volume coverage of targets were reviewed and scored (per-protocol, variation-acceptable and deviation-unacceptable) according to the protocol. We performed log-rank tests for RTQA scores with patients' outcomes, including local control (LC), distant control (DC) and overall survival (OS). Cox models with and without RTQA score data were established. To obtain a more reasonable model, per-protocol and variation acceptable were combined into a single acceptable score.

**Results:** The log-rank test showed that all RTQA scores correlated with LC, which was significantly different between the per-protocol and variation-acceptable patients in target and OAR contouring (*p*-value = 0.004 and 0.043). For dose-volume score, the per-protocol and variation-acceptable patients were significantly different from unacceptable patients in the LC, with a *p*-value = 0.020 and 0.006, respectively. The DC of patients with variation-acceptable was significantly different than that of the unacceptable patients (*p*-value = 0.043). There were no correlations between RTQA scores with other outcomes. By incorporating RTQA scores into outcome modeling, the performance of LC model can be improved from 0.62 to 0.63 (c-index). The RTQA scores had no impact on DC and OS.

**Conclusion:** RTQA scores are related to patients' local control rates in head and neck cancer radiotherapy.

## Introduction

Clinical trial quality assurance (QA) programs have been shown to be vital in ensuring that inter-institutional differences do not dilute trial results (1). In large multi-institutional trials, credible assessment of the comparative role of radiation therapy (RT) is only possible if the delivered RT is well-documented and sufficiently homogeneous in its delivery. Furthermore, it has been demonstrated that non-adherence to protocol-specified RT requirements for plan quality is associated with reduced survival and local tumor control, and can potentially lead to increased toxicity (2–7).

Most RTOG clinical trials have a radiation therapy quality assurance (RTQA) process that evaluates RT scores (contour, dose distribution) retrospectively or prospectively. Quality assurance is a resource intensive process, both from the institutions' and from the clinical trial QA centers' perspective. Furthermore, radiation therapy is a field utilizing rapidly evolving technologies such as the introduction over the last few decades of the electronic portal imaging device (EPID), the multileaf collimator (MLC), delivery technologies of intensity modulated radiation therapy (IMRT), volumetric-modulated arc therapy (VMAT) and cone-beam computed tomography (CBCT) for image guided radiotherapy (IGRT). Ensuring high quality implementation of these technologies has tremendously increased the workload for the entire radiotherapy team, and, thus, different QA procedures need to be prioritized (8). How to determine which QA methodology is relevant and efficient is of crucial significance.

With the emergence of individualized medicine and the increasing complexity (9), it is difficult to evaluate the value of one factor, which may correlate with other clinical factors. By establishing a reliable prediction model, the value of this factor can be assessed.

The aim of this study is to conduct an analysis of the correlation between RTQA scores and patient's outcome; and to evaluate the clinical value of RTQA scores by developing a quantitative predictive model of clinical outcome that contains RTQA scores and other clinical factors.

The study was performed in two parts: first we analyzed the correlation between the patient characteristics, RTQA scores and the patients' outcome; then, a logistic regression model was used to establish the prediction model. The accuracy of the model was validated by cross-validation and c-index.

## Materials and Methods

### Trial Protocol and RTQA Process

The RTOG protocol provides details of the trial design, treatment regimens (10). Briefly, patients with stage III-IV carcinoma of the oropharynx, larynx, and hypopharynx, having Zubrod performance of grade 0 to 1, and meeting predefined blood chemistry criteria were enrolled after providing informed consent. From November 2005 to March 2009, 940 patients were enrolled. After removing patients with incomplete RTQA scores data, 767 patients were enrolled in this study. All the patients passed the initial scrutiny according to the RTOG protocol. Table 1 shows patients' characteristics. Event rates at 5 years of follow-up for these patients were 80.1% for local control, 76.3% for distant control, and 66.3% for overall survival. Median follow-up times were 36.8 months for local control, 37.0 months for distant control, and 42.4 months for overall survival.

**Table 1:** Patient characteristics.

Total patients	767	100%
Age(year), median (range)	51	(31–79)
**IMRT**		
Yes	746	97.3%
No	21	2.7%
**Gender**		
Male	686	89.4%
Female	81	10.6%
**T-Stage**		
T1	7	0.9%
T2	301	39.2%
T3	282	36.8%
T4	177	23.1%
**N-Stage**		
N0	71	9.3%
N1	72	9.4%
N2	590	76.9%
N3	34	4.4%
**Primary Tumor Site**		
Oropharynx	554	72.2%
Hypopharynx	51	6.6%
Supraglottic larynx	122	15.9%
Other	40	5.2%
Hemoglobin level, mean(range)	14.3	(8–18.6)
Total radiation dose (Gy), median (range)	70	(2–73)
Total fractions, median (range)	35	(1–42)
Overall treatment time (day), median (range)	40	(1–74)
**Target Volume (TV) Contour Quality Score**		
Per-protocol	411	53.6%
Variation acceptable	310	40.4%
Deviation unacceptable	46	6.0%
**Organ At Risk (OAR) Contour Quality Score**		
Per-protocol	439	57.2%
Variation acceptable	304	39.6%
Deviation unacceptable	24	3.1%
**Target Dose-Volume Score**		
Per-protocol	490	63.9%
Variation acceptable	210	27.4%
Deviation unacceptable	67	8.7%

The case review processes (which included contour and dosimetry evaluations) were performed retrospectively by the radiation oncology and radiation physics co-chairs as described in the protocol. A quality score (per-protocol, variation acceptable and deviation unacceptable) was given to contouring and planning for major target and normal structures through the review process according to the protocol. The final overall quality score of target volume (TV_SCORE), organ at risk (OAR_SCORE) and target dose-volume coverage (TV_DVA_SCORE) is determined by the worst score in these categories. Table 2 shows the criteria for evaluation of target volume and dosimetry scores.

**Table 2:** Criteria of target volume and the dosimetry parameter.

**RT parameter**	**Per protocol**	**Variation acceptable**	**Deviation unacceptable**	**Category**
Gross Tumor Volume (GTV)	The region contains gross primary tumor or involved node(s) based on clinical and endoscopic examinations, CT scan, and other imaging techniques.	Not predefined	Not predefined	TV contour quality score
Clinical Target Volume (CTV)	GTV with a margin of 1–2 cm and nodal regions to receive elective irradiation	Not predefined	Not predefined	TV contour quality score
Planning Target Volume (PTV)	CTV with a margin of 3–5 mm	Not predefined	Not predefined	TV contour quality score
Volume of PTV receive 65 Gy	≥99%	97–99%	<97%	Target dose-volume quality score
Volume of PTV receive 70 Gy	≥95%	≥95%	<95%	Target dose-volume quality score
Volume of PTV receive 77 Gy	≤ 20%	20–40%	>40%	Target dose-volume quality score
Volume of PTV receive 80 Gy	≤ 5%	5–20%	>20%	Target dose-volume quality score

### Prognostic Factors and Correlation Analyses

The prognostic factor selection was based on Egelmeer's study (11). Clinical factors, including age at start of RT, IMRT, gender, T-stage, N-stage, primary tumor site, hemoglobin level, equivalent dose in fractions of 2Gy (EQD2) which were calculated from RT scores are selected. To simplify the model, primary tumor site was categorized into 4 groups: oropharynx supraglottic larynx, hypopharynx and others. Similarly, T-stage and N-stage were encoded into 4 ranks. EQD2 was calculated by the following formula (12):

$$\begin{array}{l} EQD_{2}=D\frac{d+\alpha /\beta }{2+\alpha /\beta }-\gamma \left(T-T_{k}\right) \end{array}$$

*D* is the total radiation dose, *d* is the fraction dose, α*/*β is 10 Gy, *T* is the overall treatment time, accelerated repopulation kick-off time (*T*_*k*_) is 28 days, and loss in dose due to repopulation (γ) is 0.6 Gy/day. After transformation, the median EQD2 is 61.6Gy (range, 20.62–65.80Gy). Among prognostic factors, age, hemoglobin level, and EQD2 were analyzed as continuous values.

Spearman correlation coefficient were calculated between clinical factors and RTQA scores. For tumor location, the chi-square test was performed to evaluate its relationship with RTQA scores. To evaluate the relationship between RTQA scores and patients' outcome, we performed log-rank tests for RTQA scores with patients' outcome. Since there are three levels for each RTQA score, the log-rank tests were performed between each two levels, including *per-protocol* vs. *variation acceptable, per-protocol* vs. *unacceptable* and *variation acceptable* vs. *unacceptable*.

### Prediction Model and Model Performance Evaluation

We used a simple modeling strategy to develop our prediction model. First, a univariate analysis was performed to select candidate (*p* < 0.05). Then, a cox model was established with these candidates. To get a reliable model performance, a 10 folder cross-validation technique was implemented. Briefly, patients were randomly separated into a training (90%) and validation dataset (10%). The model was developed in a training dataset and we assessed the performance in a validation dataset. We used c-index to evaluate model performance. To get stable results, the whole process is repeated 10 times. To get a more reasonable model, we combined *per-protocol* score and *variation acceptable* score in RTQA score into *acceptable* in modeling part. R (Version 3.3.0) was used to perform all the statistics analysis and model development.

## Results

### Correlation Analyses

**Figure 1:**
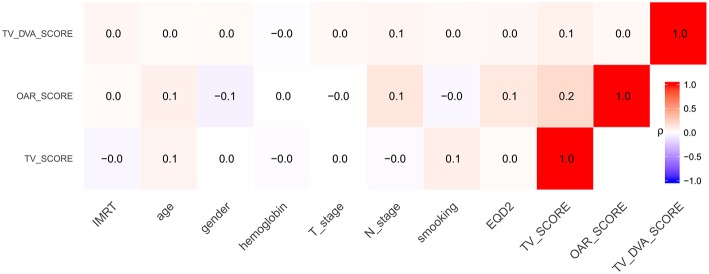
The correlation coefficient between RTQA scores with other clinical factors.

Figure 1 shows the result of the correlation analyses. The *p*-value for the chi-square test between RTQA and primary tumor site was 0.019, 0.002, and 0.147 for TV_SCORE, OAR_SCORE, and TV_DVA_SCORE, respectively.

**Figure 2:**
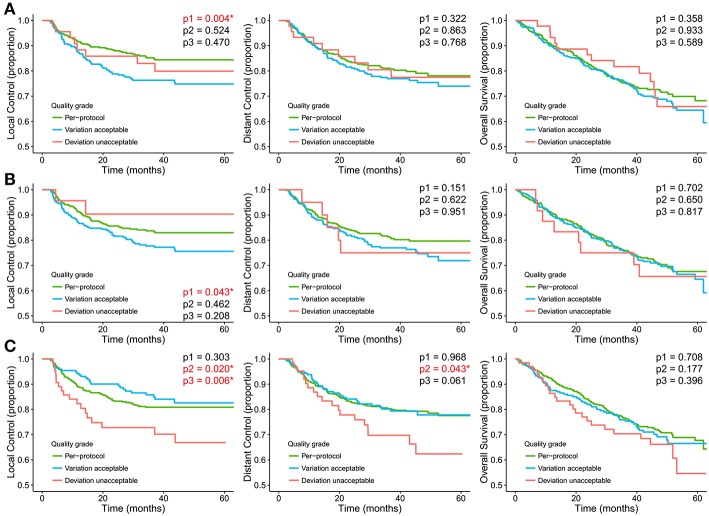
Kaplan-Meier curves stratified for the RTQA scores **(A)** Target volume (TV) contour quality score, **(B)** Organ at risk (OAR) contour quality score, **(C)** Target dose-volume score. *p1* represents the *p*-value of log-rank test between *per-protocol* and *variation acceptable*; *p2* represents the *p*-value of log-rank test between *per-protocol* and *unacceptable*; *p3* represents the *p*-value of log-rank test between *variation acceptable* and *unacceptable*. *represent *p*-value < 0.05.

Figure 2 shows the Kaplan-Meier curves for different RTQA scores. The log-rank test showed that all RTQA scores are correlated with patients' local control. For target and OAR contouring, the *per-protocol* is significantly different with *variation acceptable*, where *p*-value = 0.004 and 0.043, respectively. For dose-volume score, the *per-protocol* and v*ariation acceptable* are significantly different with *unacceptable*, where *p*-value = 0.020 and 0.006, respectively. The dose-volume score is also correlated with patients' distant control. The *variation acceptable* and *unacceptable* are significantly different, *p*-value = 0.043. There is no correlation between RTQA scores with other outcomes.

### Prediction Model and Model Validation

**Figure 3:**
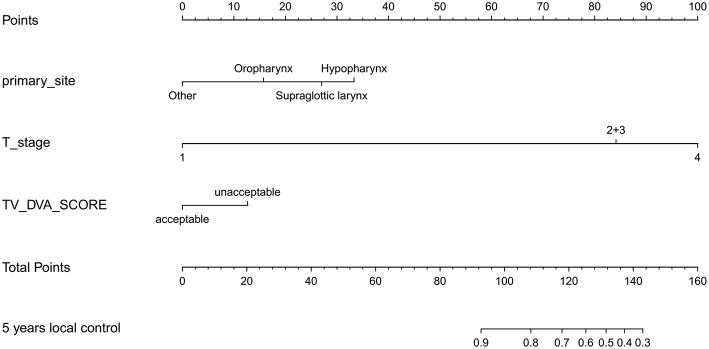
The nomogram of the local control with RTQA scores.

Table 3 shows the c-index of the prediction model. By incorporating RTQA score, the performance of the prediction model for local control was improved for 0.622 to 0.632. The RTQA scores have no impact on distant control and overall survival. Figure 3 shows the nomogram with RTQA scores for local control which demonstrates the value of RTQA scores in clinical outcomes.

**Table 3:** The c-index with or without RTQA score.

		**With RTQA score**	**Without RTQA score**
Local control	Training	0.654 [0.651 0.657]	0.635 [0.633 0.638]
	Validation	0.632 [0.619 0.645]	0.622 [0.607 0.636]
Distant control	Training	0.682 [0.680 0.684]	0.677 [0.674 0.679]
	Validation	0.652 [0.637 0.668]	0.650 [0.636 0.664]
Overall survival	Training	0.696 [0.694 0.697]	0.696 [0.695 0.698]
	Validation	0.673 [0.661 0.685]	0.675 [0.664 0.685]

## Discussion

In this study, the relationship between the RTQA scores and clinical outcomes were analyzed and the value of the RTQA scores was evaluated by prediction models. The results showed that the qualities of contouring and treatment plan are correlated to patient local control. Further analysis demonstrated only dose-volume score can be used as an independent factor for patient's local control prediction. Although, dose-volume score is correlated with patient distant control, there is no clinical value for this score in patient's distant control prediction.

**Figure 4:**
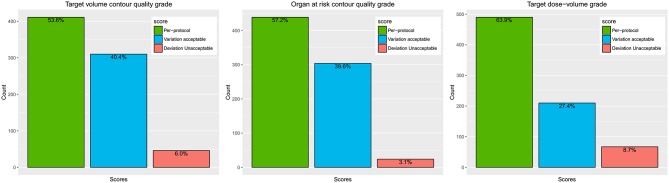
The distribution of the RTQA scores.

RTQA criteria has direct impact on the final RTQA score, especially for the dosimetry evaluation. Strict criteria will increase the plan difficulty and decrease the ratio of per protocol plans. For example, in this study, 63.9% patients belong to *per-protocol* of target dose-volume score. If we use a more loose criteria such as *variation acceptable*, 91.3% patients will belong to this category (Figure 4). How to find appropriate QA criteria is of utmost importance and remains a significant challenge. It would be better to analyze enough cases before defining the *per-protocol* and *variation acceptable* limits.

We grouped *per-protocol* and *variation acceptable* into one category in modeling base on the original ideal of the quality score. The original purpose of the quality score in the protocol was to provide a mechanism for stating the prescription for normal situations and more difficult treatment planning situations; the *per-protocol* criterion is used to encourage institutions to devise treatment plans that are as tight as possible in terms of dose conformity for PTV coverage. The *variation acceptable* compliance criterion is given to allow leeway for more difficult treatment planning situations. The *deviation unacceptable* is used to indicate incorrect prescription (13). However, this combination may decrease the model performance for prediction modeling. As Figure 4 shows, *deviation unacceptable* only has a few patients, especially for contour quality score (6.0 and 3.1%). This may cause some bias also in statistics analysis; the log-rank test shows that Kaplan-Meier curves are significantly different for contour score *per-protocol* and *variation acceptable*. However, the *unacceptable* group is not significantly different from other groups. Obviously, it was not reasonable. This bias could be corrected by including more data.

For dose-volume score, the target dose-volume coverage low quality score was caused mainly by two factors. For one, the geometry of the case makes radiotherapy planning so difficult that it is impossible to achieve a *per-protocol* plan (e.g., the target volume may have a large overlap with organ at risk or technology limitations that do not allow extremely rapid dose falloff). These are the cases that would be scored as *variation acceptable*. The other possible explanation is the planner may not have sufficient experience and skills to find an acceptable plan. The TROG study shows the quality of radiotherapy is most highly correlated with the number of patients enrolled at each center (3). This speaks to both the issue of experience and skill and the issue of available advanced technologies. To distinguish these two factors, further investigation is necessary.

In this study, we used the criteria defined in the protocol. The target dose coverage, minimum dose and maximum dose were included in the evaluation criteria. This trial started in 2006 when the IMRT technology had not been implemented fully, the reason OAR dosimetry quality score is not recorded. For the same reason, two RTQA parameters (OAR contour quality score and Target dose-volume score) were not recorded if the patient was not treated with IMRT in this study.

There were few patients who fell into the “deviation unacceptable” category, making statistical correlation with this outcome measure difficult. One valuable study is to repeat the analysis in another large dataset in which there was a higher percentage of cases with unacceptable deviations. Abrams et al. (7) also investigated the impact of adherence to specified RT protocol guidelines on protocol outcomes for pancreatic adenocarcinoma. They found that failure to adhere to specified RT guidelines was associated with reduced survival and, for patients receiving gemcitabine, trend toward increased non-hematologic toxicity. In our study, we did not analysis the impact of RTQA score on radiotherapy toxicity. Further, both of the above analyses will be performed in future studies.

Although the experiment using current indexes demonstrated a promising result, there is a big challenge of the very subjective nature of both contouring and assessment of contours. The cases from multi-center have large deviation in contouring due to the inter-observe variation. Similarly, for contour quality score, these quality scores also depend mainly on the physicians' subjective judgment. Moreover, the scoring has only three levels. Therefore, more objective and quantitative criteria is needed for contour assessment. Quantitative evaluation of contours may become more feasible in the future with technology developments in the areas of functional imaging, deformable registration, and contour atlas. We are working on this and will attempt to analyze more clinical trials to investigate the influence of RTQA score on treatment outcome.

## Conclusions

This exploratory analysis found that the RTQA scores were related to patient local control in RTOG 0522 trial. The influence of the subjective nature of quality scoring remains unknown. A more reasonable controlled trial with objective and pre-designed quality index merits further investigation.

## Data Availability

The datasets generated and/or analyzed during the current study are not publicly available according to the Data Sharing Policy of NRG Oncology. Requests to access the datasets should be directed to [NRG Oncology, APC@nrgoncology.org].

## Ethics Statement

This retrospective study was approved and carried out in accordance with the Data Sharing Policy of NRG Oncology. All the data are de-identified. In addition, based on the retrospective character of this analysis, no additional written informed consent was needed.

## Author Contributions

All authors discussed and conceived of the study design. HZ wrote the programs and performed data analysis and drafted the manuscript. KM, JW, JvS, DR, AD, and ZZ discussed and made suggestions. YX guided the study and participated in discussions and preparation of the manuscript. All authors read, discussed, and approved the final manuscript.

### Conflict of Interest Statement

The authors declare that the research was conducted in the absence of any commercial or financial relationships that could be construed as a potential conflict of interest.

## Abbreviations

RTQA: radiation treatment quality assurance
TV: target volume
OAR: organ at risk
LC: local control
DC: distant control
OS: overall survival
QA: quality assurance
RT: radiation therapy
EPID: electronic portal imaging device
MLC: multileaf collimator
IMRT: intensity-modulated radiotherapy
VMAT: volumetric-modulated arc therapy
CBCT: cone-beam computed tomography
IGRT: image guided radiotherapy
TV_SCORE: score of target volume
OAR_SCORE: score of organ at risk
TV_DVA_SCORE: score of target dose-volume coverage
GTV: gross tumor volume
CTV: clinical target volume
PTV: planning target volume
EQD2: equivalent dose in fractions of 2Gy.
